# MicroRNA‐302c‐3p inhibits endothelial cell pyroptosis via directly targeting NOD‐, LRR‐ and pyrin domain‐containing protein 3 in atherosclerosis

**DOI:** 10.1111/jcmm.16500

**Published:** 2021-03-30

**Authors:** Baochen Bai, Yanyan Yang, Shengxiang Ji, Shizhong Wang, Xingang Peng, Chao Tian, Rui‐Cong Sun, Tao Yu, Xian‐ming Chu

**Affiliations:** ^1^ Department of Cardiology The Affiliated Hospital of Qingdao University Qingdao China; ^2^ Department of Immunology Basic Medicine School Qingdao University Qingdao China; ^3^ Department of Microbiology Linyi Center for Disease Control and Prevention Linyi China; ^4^ Department of Cardiovascular Surgery The Affiliated Hospital of Qingdao University Qingdao China; ^5^ The Department of Emergency General Surgery The Affiliated Hospital of Qingdao University Qingdao China; ^6^ Department of Cardiac Ultrasound The Affiliated hospital of Qingdao University Qingdao China; ^7^ Institute for Translational Medicine The Affiliated Hospital of Qingdao University Qingdao China; ^8^ Department of Cardiology The Affiliated Cardiovascular Hospital of Qingdao University Qingdao China

**Keywords:** atherosclerosis, endothelial cell, miR‐302c‐3p, NLRP3, pyroptosis

## Abstract

Inflammation and endothelial dysfunction are important participants and drivers in atherosclerosis. NOD‐, LRR‐ and pyrin domain‐containing protein 3 (NLRP3) inflammasome activation and the resulting pyroptosis are involved in the initiation and vicious circle of chronic inflammation, thus playing an indispensable role in atherosclerosis. Accordingly, blocking the activation of NLRP3 inflammasome may be a promising treatment strategy to blunt the progression of atherosclerosis. In this study, it was demonstrated that miR‐302c‐3p exerted anti‐pyroptosis effects by directly targeting NLRP3 in vivo and in vitro. In brief, the expression of miR‐302c‐3p was down‐regulated whereas the expression of NLRP3 was up‐regulated in human plaques and in vitro pyroptosis model of endothelial cells. Overexpression of miR‐302c‐3p suppressed endothelial cell pyroptosis by targeting specific sites of NLRP3. By comparison, down‐regulation of endogenous miR‐302c‐3p led to the opposite results, which were reversed by silencing the expression of NLRP3. Finally, the up‐regulation of miR‐302c‐3p inhibited the inflammation and pyroptosis of atherosclerosis mouse model. In conclusion, miR‐302c‐3p may be a powerful and attractive target for suppressing endothelial inflammation and pyroptosis, providing a novel strategy for preventing or alleviating the progression of atherosclerosis.

## 1 INTRODUCTION

Atherosclerosis (AS) is a chronic inflammatory disease accompanied by lipid accumulation and endothelial dysfunction.^1^, ^2^, ^3^ Early identification is essential in subclinical atherosclerosis because of a remarkably long period of incubation and the close association with cardiovascular diseases.^4^ Oxidized low‐density lipoprotein (ox‐LDL) is a key factor in the progression of atherosclerotic cardiovascular disease.^5^ Briefly, ox‐LDL is recognized by pattern recognition receptors in the endothelium and induces endothelial dysfunction by triggering a cascade of oxidative stress and inflammatory responses, which are characteristics of atherosclerosis.^5^, ^6^ In this sense, the prompt intervention of endothelial inflammation and immunity would delay atherosclerosis.

NLRP3 inflammasome, a multimeric protein receptor, acts as a cytosolic innate immune signalling platform and a key mediator of inflammation and immunity,^7^, ^8^, ^9^ which is formed by the sensor protein NLRP3, the adaptor ASC [apoptosis­associated speck‐like protein containing a CARD (caspase recruitment domain)] and the effector caspase‐1. Its activation recruits pro‐caspase‐1 and triggers autoproteolytic cleavage into activated caspase‐1, resulting in the cleavage of gasdermin D (GSDMD) and membrane rupture with an inflammatory programmed cell death, termed pyroptosis. Concomitantly, inactive pro‐interleukin (IL)‐1β and pro‐IL‐18 are cleaved by caspase‐1 into active IL‐1β and IL‐18.^10^ Duewell et al have found that NLRP3 inflammasome is triggered and activated by cholesterol crystals, resulting in chronic inflammation and the formation of atherosclerotic plaques, which unravelled the pivotal role of the NLRP3 inflammasome in atherosclerosis.^11^ As then, the mechanism of NLRP3 inflammasome involvement in atherosclerosis has been extensively studied in immune cells, including monocytes and macrophages.^12^ However, recently, increasing studies have reported that the NLRP3 inflammasome also exerts a essential role in endothelial cells.^13^ Zhuang et al have demonstrated that endothelial Forkhead box P transcription factor 1 attenuates endothelial inflammation by directly inhibiting NLRP3 inflammasome activation.^14^ Moreover, Zhang *et al* have found that melatonin exerts anti‐pyroptosis effects in endothelial cells, which is achieved by regulating the long non‐coding RNA maternally expressed 3/miR‐223/NLRP3 axis.^15^ Directly inhibiting NLRP3 inflammasome activation not only inhibits the secretion of IL‐18 and IL‐1β, but also inhibits the occurrence of pyroptosis, further reducing the cascade of local inflammation. An increasing number of NLRP3 inflammasome inhibitors have been discovered, such as MCC950,^16^ b‐hydroxybutyrate (BHB)^17^ and OLT1177,^18^ which block NLRP3 inflammasome activation and attenuate IL‐18 and IL‐1β release, thereby alleviating inflammatory diseases. Therefore, NLRP3 may be an effective therapeutic target for cardiovascular disease.

Non‐coding RNAs (ncRNAs) are novel regulators of cell function and disease progression.^19^, ^20^ Recently, accumulating evidence indicates that ncRNAs play a critical regulatory role in cardiovascular disease.^21^, ^22^, ^23^, ^24^, ^25^, ^26^, ^27^ MicroRNAs (miRNAs) are one of the most thoroughly studied and highly conserved ncRNAs, which are 20‐25 nucleotides in length and impede gene expression via silencing their target mRNAs post‐transcriptionally, thus involving a variety of critical cellular processes and diseases.^28^, ^29^, ^30^ miRNAs regulate endothelial cell functions by acting as sensors of inflammation and protectors of endothelial homeostasis.^31^ In this regard, emerging literature suggests that miRNAs also fine‐tuning the process of NLRP3 inflammasome activation, which is accomplished by lowering NLRP3 expression at the translational level.^32^, ^33^, ^34^ However, which miRNAs have the most significant impact on NLRP3 function in the cardiovascular field remains to be elucidated.

In this study, miRNAs directly targeting NLRP3 were screened and identified using an online bioinformatics database and the most significant, miR‐302c‐3p, was selected through a variety of experimental verifications. miR‐302c‐3p diminished endothelial inflammation and pyroptosis by directly binding to the specific sites of NLRP3 mRNA. Additionally, the aortic inflammation and pyroptosis of ApoE^‐/‐^ mice were ameliorated after miR‐302c‐3p agomir treatment via tail vein injection. These results demonstrated a novel miR‐302c‐3p/NLRP3 regulatory signalling pathway and the underlying mechanism in endothelial cells.

## 2 MATERIALS AND METHODS

### 2.1 Study subjects

All atherosclerotic arterial samples of patients undergoing carotid endarterectomy and control arterial tissues of individuals who died in traffic accidents without obvious atherosclerosis were provided from The Affiliated Hospital of Qingdao University. All tissue samples were stored in liquid nitrogen until use. All clinical samples were obtained with the informed consent of patients and the ethical approval of the Ethical Committee of the Affiliated Hospital of Qingdao University.

### 2.2 Bioinformatics analysis and miRNA screening

The upstream miRNAs targeting NLRP3 were predicted by four bioinformatics websites: miRmap (https://mirmap.ezlab.org/app/), Targetscan (http://www.targetscan.org/vert_72/), miRWalk (http://mirwalk.umm.uni‐heidelberg.de/) and miRanda (http://mirdb.org/). Then, the collection of miRNAs was obtained using the Venn diagram online production website (http://bioinformatics.psb.ugent.be/webtools/Venn/). Through comprehensively evaluating the website prediction scores, miRNA conservative scores and the degree of correlation with cardiovascular inflammation, 4 miRNAs were identified from 21 miRNAs in 4 databases, namely miR‐302c‐3p, miR‐490‐5p, miR‐421 and miR‐876‐5p. Eventually, miR‐302c‐3p was chosen as the research subject because of the negative correlation with the expression of NLRP3 in human plaque tissues.

### 2.3 Atherosclerosis model establishment and treatment

Eight‐week‐old ApoE^‐/‐^ and wild‐type (WT) male C57BL/6J mice were procured from the Laboratory Animal Center of Vital River (Beijing, China). All animals were housed under standard conditions where water and food are freely available. For the atherosclerosis model, ApoE^‐/‐^ mice, as a high‐fat diet (HFD) group, were given a HFD containing 2% cholesterol for 12 weeks (n = 14). By comparison, WT mice, as a normal diet (ND) group, received a ND under the same conditions (n = 7). After feeding the mice for 12 weeks, seven mice were randomly selected from the HFD group as the treatment group. For the in vivo miRNA delivery experiment, mice were injected with miR‐302c‐3p agomir mixed with polyethylenimine‐graft‐poly(ethylene glycol) (PEI‐*g*‐PEG) copolymers through the tail vein (1 mg/kg, twice a week for 4 weeks).^35^ All experiments were performed after obtaining the approval of the Animal Ethics Committee of the Affiliated Hospital of Qingdao University (Shandong, China).

### 2.4 Analysis of atherosclerotic lesions

After the last miR‐302c‐3p agomir treatment, chloral hydrate was used to anaesthetize the mice by intraperitoneal injection. Then, the aorta was separated from the heart to the iliac bifurcation, and the adventitia tissue was carefully removed. The aortic root tissue was immediately cross‐cut into 6 μm thick slices using a frozen section machine and stored at −80°C. The aorta frozen sections were stained with 0.5% oil red o for 15 min, then destained with 60% isopropanol three times, 5 min each, and finally washed twice with distilled water. The lipid staining of arterial plaque was visualized using a microscope (Olympus, Tokyo, Japan) and quantified with ImageJ software.

### 2.5 Cell culture and treatment

HUVECs were obtained from Cell Bank, Chinese Academy of Sciences (Shanghai, China) and cultured in DMEM/F‐12 medium (Meilunbio, Dalian, China) containing 10% fetal bovine serum (ExCell Bio, Shanghai, China) and placed in a standard cell incubator. When the cell density reached about 80%, HUVECs were seeded into 6‐ or 12‐well plates and treated with ox‐LDL (100 µg/mL) (Meilunbio, Dalian, China) for 24 h to stimulate and induce pyroptosis.

### 2.6 Cell transfection and grouping

The miR‐302c‐3p mimic, miR‐302c‐3p inhibitor and small‐interfering RNAs targeting NLRP3 (si‐NLRP3) were synthesized by GenePharma (Shanghai, China). HUVECs were transfected using the transfection reagent Lipofectamine 3000 (Invitrogen), according to the manufacturer's protocol for 24 h when cells reached 80% confluence. After that, the medium was renewed and cells were treated with or without ox‐LDL. HUVECs were classified into the miR‐302c‐3p mimic group, miR‐302c‐3p inhibitor group, negative control (NC) group, inhibitor NC (in‐NC) group, si‐NLRP3 group and si‐NC group. The sequences required for transfection are listed in Table S1.

### 2.7 Quantitative real‐time polymerase chain reaction (qRT‐PCR)

The RNA of aortic tissues and HUVECs were extracted using TRIzol reagent (Sigma, St. Louis, MO, USA), following the manufacturer's protocols. Subsequently, 1 µg RNA was reverse‐transcribed into cDNA using the Mir‐X miRNA First‐Strand Synthesis Kit or PrimeScript RT reagent Kit with gDNA Eraser (TaKaRa, Dalian, China), respectively. Then, SYBR Green PCR Master Mix (Yeasen, Shanghai, China) was used to perform qRT‐PCR. Table S2 lists the PCR primers used in the experiment, which were purchased from TsingKe (Beijing, China). U6 was considered as internal reference of miRNA, and GAPDH was an housekeeping gene to other target genes. The relative gene expression was analysed using the 2^−△△Ct^ method.

### 2.8 Western blots

Radio immunoprecipitation assay (RIPA) lysis buffer (EpiZyme, Shanghai, China) was used to extract total protein from aortic tissues and HUVECs. The protein concentration was measured using the BCA detection method. Subsequently, the equivalent amount of protein was separated by 12.5% SDS‐PAGE, then wet transferred to PVDF membranes (Millipore, Boston, MA, USA) and blocked with 5% non‐fat milk for 1 h at 37°C, followed by incubated overnight at 4°C with the rabbit anti‐NLRP3 antibody (1:1000, ab263899, Abcam, MA, USA), rabbit anti‐cleaved caspase‐1 p20 antibody (1:2000, AF4005, Affinity, OH, USA), rabbit anti‐cleaved‐IL‐1β antibody (1:2000, AF4006, Affinity, OH, USA), rabbit anti‐GSDMD antibody (1:2000, AF4012, Affinity, OH, USA) or anti‐β‐actin antibody (1:1000, #4967, Cell Signaling Technology, USA). β‐actin served as the housekeeping protein control. After washing with TBST three times, the membranes were incubated with the relevant HRP‐conjugated secondary antibody at 37°C for 1 h. The chemiluminescence detection kits (Meilunbio, Dalian, China) were used to visualize protein bands. Finally, ImageJ software was used for densitometric analysis.

### 2.9 Biotinylated miRNA pull‐down assay

A biotin‐labelled miR‐302c‐3p pull‐down assay was performed, as previously described.^36^ In brief, biotin‐labelled miR‐302c‐3p (Beijing Genomics institution, Beijing, China) was transfected into HUVECs for 24 h. Then, the cells were lysed, then sonicated and centrifuged. The supernatant (50 μL) was separated and used as input. After pre‐blocking the beads with 1% RNase‐free bovine serum albumin and 0.5 mg/mL yeast tRNA (Sigma), the remaining lysates were incubated with MyOne Streptavidin C1 Dynabeads (Invitrogen, CA, USA) for 3 h at 4°C. The beads were then thoroughly washed in wash buffer. Finally, the bound RNA was extracted using TRIzol and detected by qRT‐PCR.

### 2.10 Dual luciferase reporter gene assay

A fragment of the WT or mutant (Mut) sequences containing the binding sites of miR‐302c‐3p and NLRP3 was, respectively, inserted between the SacI and SalI restriction sites in the pmirGLO vector (Promega). WT or Mut recombinant plasmids with miR‐302c‐3p mimic or NC were cotransfected in 293T cells using Lipofectamine 3000 for 24 h. A Firefly & Renilla‐Glo Luciferase Reporter Assay Kit (Meilunbio, Dalian, China) was used to detect the luciferase activity.

### 2.11 Lactate dehydrogenase (LDH) release assay

An LDH release assay was used to detect the LDH activity in the cell supernatant after pyroptosis. The LDH activity was evaluated from the supernatant of the treated cells using a LDH assay kit (Nanjing Jiancheng, Jiangsu, China) following the instructions.

### 2.12 Hoechst 33342/propidium iodide (PI) staining

HUVECs were seeded into 24‐well plates with varying treatments, and then, cells were stained using the Hoechst 33342/PI double staining kit (Meilunbio, Dalian, China). Briefly, an equal amount of matrix buffer was added to cells, followed by incubation with Hoechst 33 342 (5 µL) and PI (5 µL) solutions at 4°C without light for 20‐30 min. The stained cells were analysed using a Olympus fluorescence microscope and quantified by ImageJ software.

### 2.13 Immunofluorescence staining

The aortic root tissue was immediately embedded in OCT compound and then use a frozen section machine to cross‐cut it into 6 μm thick slices. Before staining, 0.5% Triton X‐100 was used to permeabilize the frozen sections for 10 min. Subsequently, the sections were blocked with 10% rabbit serum for 30 min and incubated with anti‐NLRP3 antibody (1:100, DF7438, Affinity, OH, USA) for 2 h, then incubated with fluorescent secondary antibodies (1:100, 111‐095‐003, Jackson ImmunoResearch, PA, USA) for 1 h in the dark. Next, DAPI was dropped on the sections to stain the nuclei for 5 min. Finally, the sections were analysed by a Olympus fluorescence microscope and quantified by imagej software.

### 2.14 Immunohistochemical assay

The mouse aortic tissues were immersed in formalin for 48 h, then dehydrated by gradient ethanol and vitrified by dimethylbenzene. Subsequently, paraffin‐embedded tissue sections (4 μm thick) were deparaffinized and rehydrated and then incubated with sodium citrate buffer to repair the antigen. Next, the blocked sections were incubated with anti‐NLRP3 antibody (1:100, DF7438, Affinity, Cincinnati, OH, USA) and then incubated with HRP‐conjugated secondary antibodies (Zhongshan Golden Bridge, Beijing, China) for 30 min, and then visualized using newly prepared 3,3'‐diaminobenzidine for 1 min. After the sections were washed and counterstained with haematoxylin for 1 min, they were observed with a microscope (Olympus, Tokyo, Japan).

### 2.15 RNA fluorescence in situ hybridization (FISH)

The cy3‐labelled miR‐302c‐3p probe (5ʹ‐CCACTGAAACATGGAAGCACTTA‐3ʹ) was obtained from GenePharma (Shanghai, China). The frozen section of the aortic root was infiltrated in potassium chloride hypotonic solution for 40 min and then fixed with fixative for 40 min. Subsequently, it was placed in a thermostat at 56°C for 20 min and treated with compound digestive solution containing pepsin and proteinase K for 10 min. The sections were pre‐denatured by incubating with probe hybridization solution at 75°C for 7 min and then hybridized at 40°C overnight in a dark and humid environment. After washing with saline‐sodium citrate solution, DAPI was added to stain the nucleus for 5 min. Finally, the sections were observed with a confocal microscope (Leica, Hesse, Germany).

### 2.16 Statistical analysis

GraphPad Prism 5 software was used for analysing all data in this study. The data were shown as mean ± standard error of the mean (SEM). The comparisons between independent groups were conducted with two tailed Student's t test. Statistically significant was defined by *P* value < .05.

## 3 RESULTS

### 3.1 Screening of miRNAs directly targeting NLRP3

**FIGURE 1:**
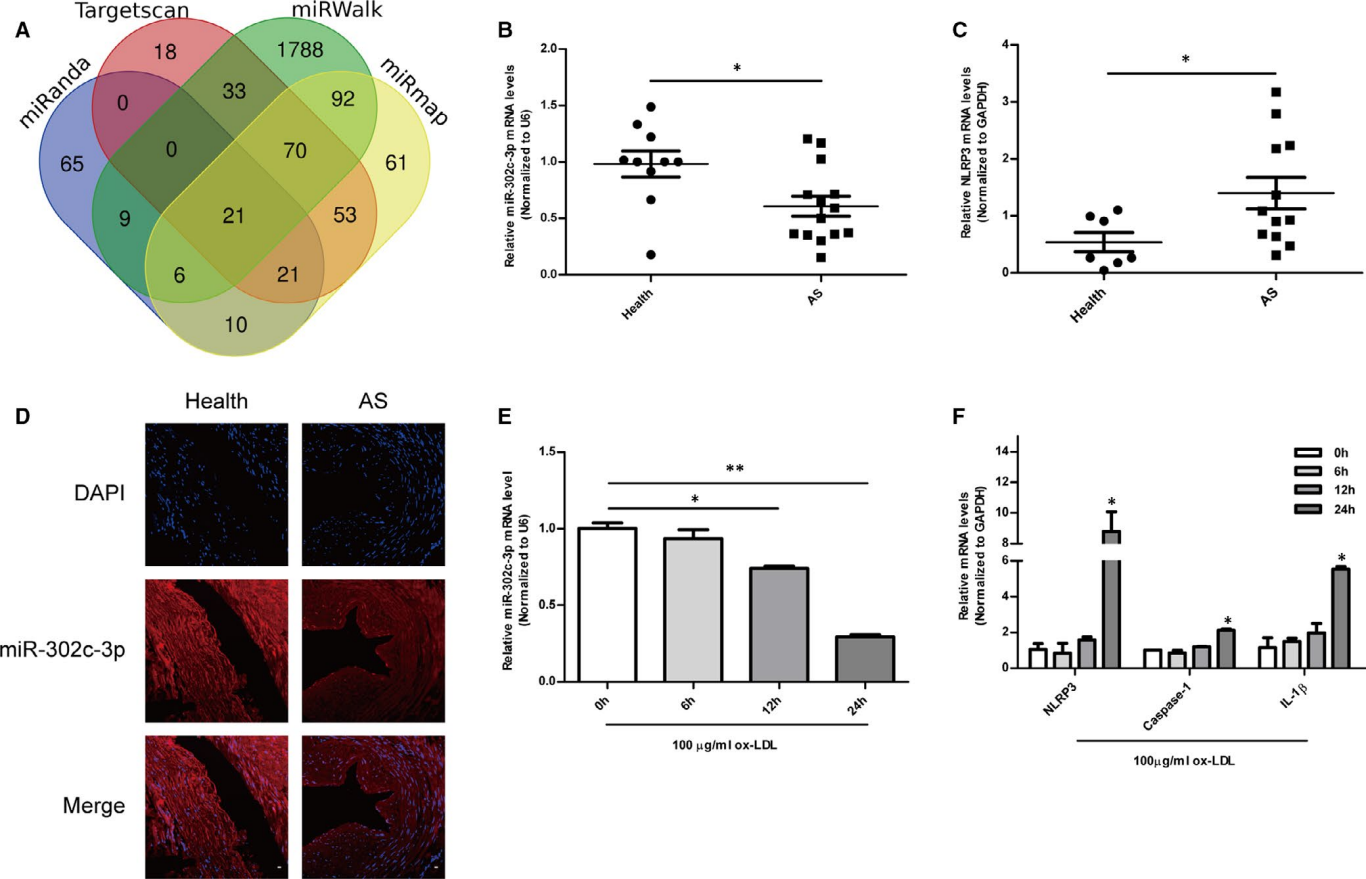
Screening of miRNAs directly targeting NLRP3. A, Venn diagram of four independent miRNA differential analysis databases reveals 21 possible miRNAs directly targeting NLRP3. B, qRT‐PCR analysis of miR‐302c‐3p levels in healthy arterial tissues (n = 10) and atherosclerotic arterial samples (n = 14). C, qRT‐PCR analysis of NLRP3 levels in healthy arterial tissues (n = 7) and atherosclerotic arterial tissues (n = 12). D, miR‐302c‐3p expression in human normal and atherosclerotic arteries, as detected by FISH. E, qRT‐PCR analysis of miR‐302c‐3p expression in HUVECs treated with 100 µg/mL ox‐LDL at the indicated times (0, 6, 12 and 24 h). F, qRT‐PCR analysis of NLRP3, caspase‐1, and IL‐1β expression in HUVECs treated with 100 µg/mL ox‐LDL at the indicated times (0, 6, 12 and 24 h). Scale bars: 50 μm. *P < .05; **P < .01; ***P < .001. Error bars indicate the mean ± SEM of at least triplicate independent experiments

miRNAs that directly targeted NLRP3 were screened using four bioinformatics websites: miRmap, Targetscan, miRWalk and miRanda. Twenty‐one miRNAs were identified in the intersection using online Venn diagram analysis (Figure 1A). Through comprehensively evaluating the website prediction scores, miRNA conservative scores, and the degree of correlation with cardiovascular inflammation, 4 miRNAs from 21 miRNAs in 4 databases were identified, namely miR‐302c‐3p, miR‐490‐5p, miR‐421 and miR‐876‐5p. Next, the expression levels of the four miRNAs and NLRP3 were tested in human normal arterial tissues and plaque tissues. miR‐302c‐3p expression was negatively correlated with NLRP3 expression, whereas the expression of other three miRNAs was positively correlated with NLRP3 expression by qRT‐PCR (Figure 1B,C and Figure S1A–C). Hence, it was speculated that miR‐302c‐3p was a potential miRNA targeting NLRP3. RNA FISH analysis of miR‐302c‐3p also showed low expression of miR‐302c‐3p in human plaque tissues compared to that in healthy tissues (Figure 1D). Ox‐LDL‐treated HUVECs were used as an in vitro NLRP3 inflammasome activation cell model to explore the association between miR‐302c‐3p and NLRP3. As shown in Figure 1E and F, miR‐302c‐3p expression is also negatively correlated with NLRP3 inflammasome‐related cytokines (NLRP3, IL‐1β and caspase‐1) in ox‐LDL‐treated HUVECs. Thus, data from clinical samples to cell models suggested a potential link between NLRP3 and miR‐302c‐3p.

### 3.2 MiR‐302c‐3p directly targeted and inhibited NLRP3 expression

**FIGURE 2:**
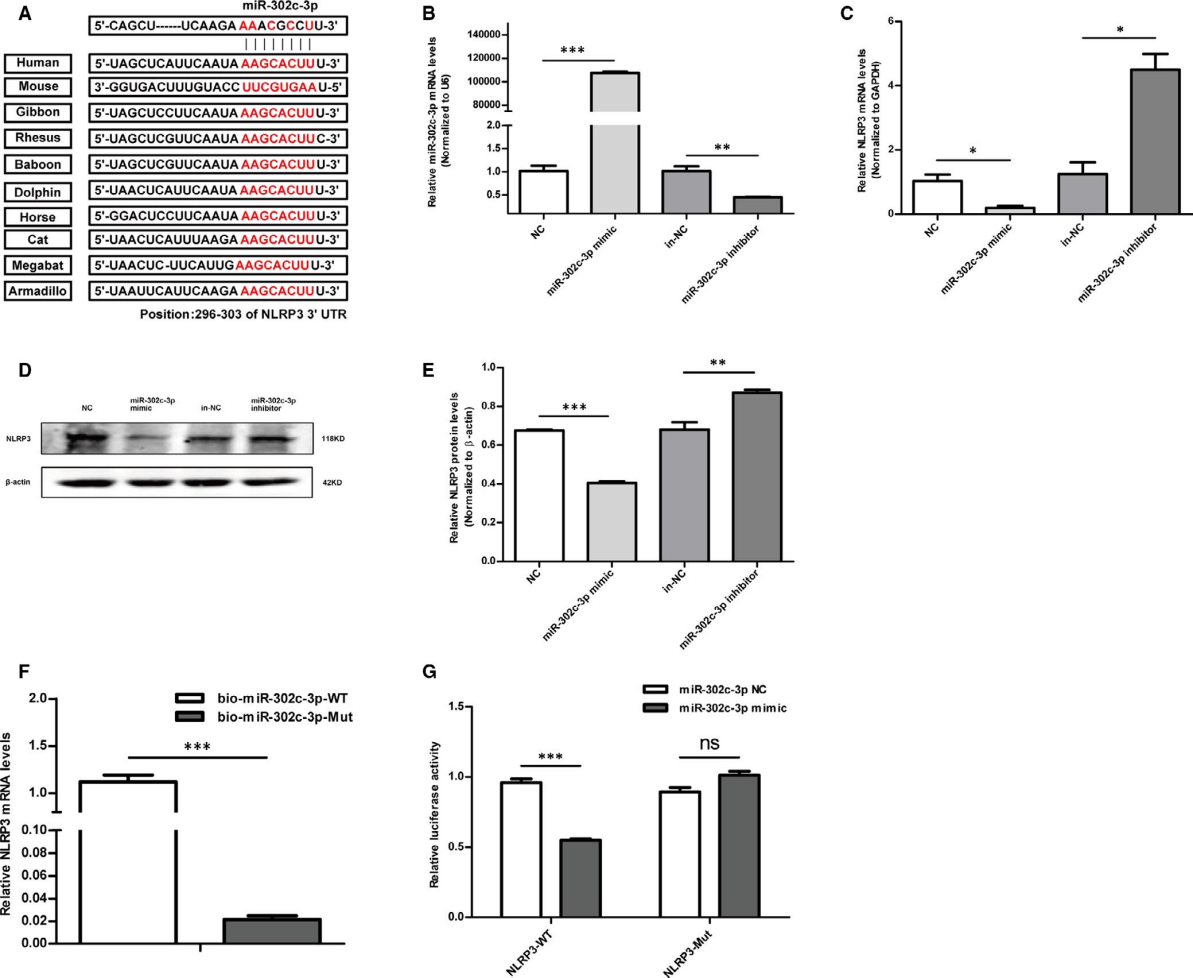
MiR‐302c‐3p directly targets and inhibits NLRP3 expression. A, Predicted miR‐302c‐3p target sequences in the NLRP3 3′ UTR of different species. B, The transfection efficiency of miR‐302c‐3p is shown, as detected by qRT‐PCR. C‐E, qRT‐PCR and Western blot analysis of NLRP3 expression in HUVECs transfected with the miR‐302c‐3p mimic, miR‐302c‐3p mimic negative control (NC), miR‐302c‐3p inhibitor and miR‐302c‐3p inhibitor NC (in‐NC). F, Expression levels of NLRP3 are shown, as tested by qRT‐PCR after biotinylated miR‐302c‐3p or its mutant were transfected into HUVECs for 24 h. G, Luciferase activity in 293T cells cotransfected with the miR‐302c‐3p mimic or miR‐302c‐3p NC and NLRP3 3′ UTR wild‐type (WT) or mutant (Mut) recombinant plasmids containing the miR‐302c‐3p binding sites. *P < .05; **P < .01; ***P < .001. Error bars indicate the mean ± SEM of at least triplicate independent experiments

To ascertain whether miR‐302c‐3p directly targeted NLRP3, the binding sites between miR‐302c‐3p and NLRP3 were predicted and were highly conserved in primates and mammals (Figure 2A). Subsequently, the mimic and inhibitor of miR‐302c‐3p were synthesized and transfected into HUVECs, respectively, and the transfection efficiency was tested (Figure 2B). It was observed that the mRNA expression of NLRP3 was decreased in HUVECs transfected with the miR‐302c‐3p mimic, whereas increased in the group of transfection with miR‐302c‐3p inhibitor (Figure 2C). NLRP3 protein expression was also found to have the same results (Figure 2D,E). Biotin‐labelled miR‐302c‐3p and its mutant mimics were used to test whether miR‐302c‐3p could pull down NLRP3 in HUVECs. After transfection, HUVECs were harvested to extract enriched RNA for a pull‐down assay. A higher enrichment of NLRP3 was observed in the captured fraction of the biotin‐miR‐302c‐3p‐WT group compared to that in the biotin‐miR‐302c‐3p‐Mut group (Figure 2F). Subsequently, a luciferase reporter gene test was performed. The NLRP3‐WT or NLRP3‐Mut recombinant plasmids containing the binding sites of miR‐302c‐3p were cotransfected with the miR‐302c‐3p mimic or NC into 293T cells. As a result, luciferase activity was suppressed in the group of cotransfection of NLRP3‐WT and miR‐302c‐3p mimic. However, cotransfection of miR‐302c‐3p mimic and NLRP3‐Mut did not affect luciferase activity (Figure 2G). Collectively, the above data demonstrated that miR‐302c‐3p inhibited the expression of NLRP3 by directly binding to specific miR‐302c‐3p binding sites.

### 3.3 Overexpression of miR‐302c‐3p reversed ox‐LDL‐induced pyroptosis in HUVECs

**FIGURE 3:**
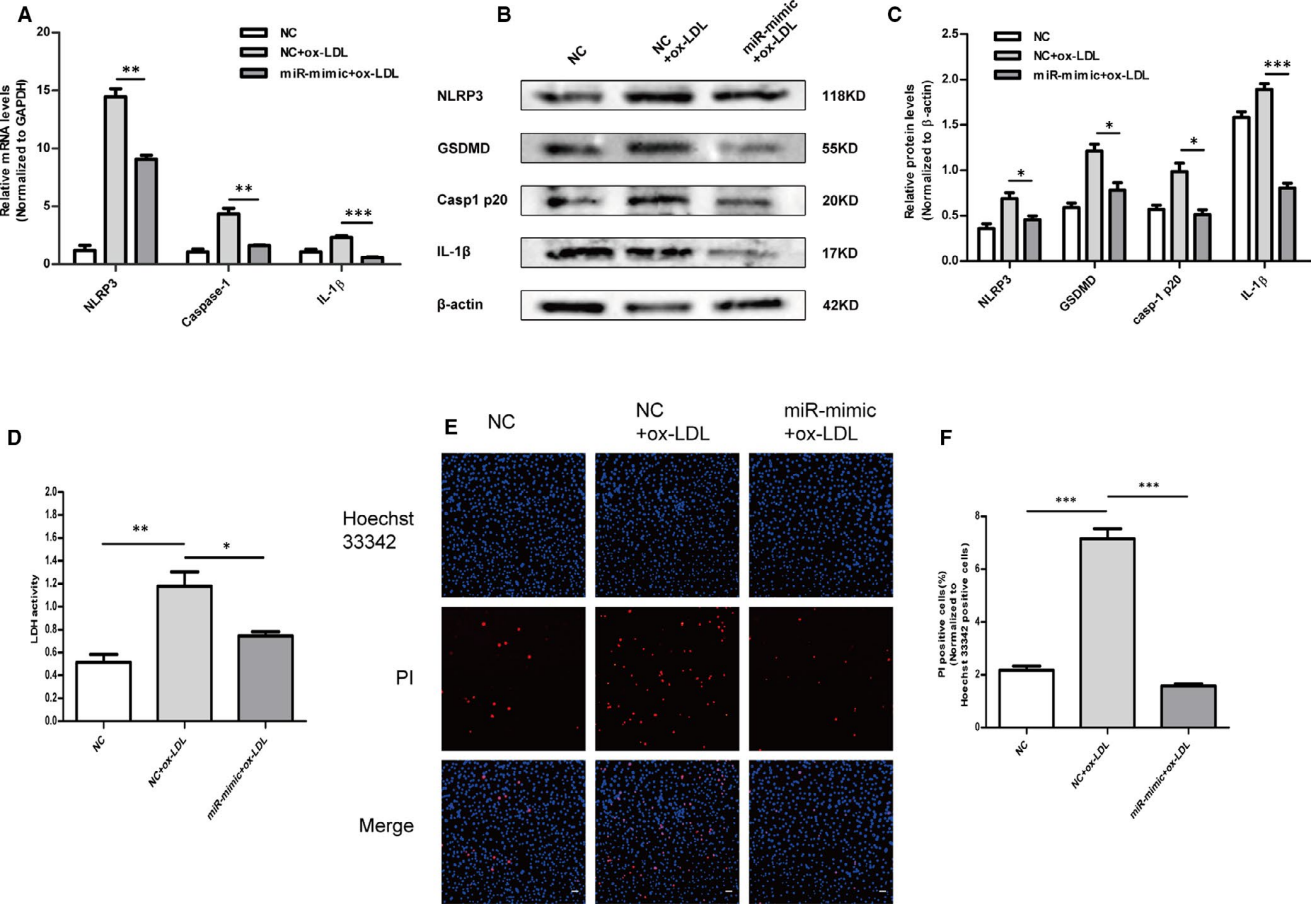
Overexpression of miR‐302c‐3p reverses ox‐LDL‐induced pyroptosis in HUVECs. HUVECs were transfected with the NC or miR‐302c‐3p mimic and then treated with 100 µg/mL ox‐LDL. A, qRT‐PCR analysis of NLRP3, caspase‐1 and IL‐1β expression. B and C, Western blot analysis and quantification of NLRP3, GSDMD, casp1 p20 and IL‐1β expression. D, Lactate dehydrogenase (LDH) release assay detecting LDH activity in the cell supernatant. E, Representative fluorescence micrographs of cells stained with propidium iodide (PI, red) and Hoechst 33 342 (blue). F, Quantification of the percentage of PI‐positive cells. PI‐positive cells are reduced in the miR‐302c‐3p mimic + ox‐LDL group compared to those in the NC + ox‐LDL group. Scale bars: 50 μm. *P <.05; **P <.01; ***P <.001. Error bars indicate the mean ± SEM of at least triplicate independent experiments

Pyroptosis is an inflammatory programmed cell death resulting from NLRP3 inflammasome activation.^37^ Thus, whether the abnormal expression of miR‐302c‐3p regulated endothelial cell pyroptosis by targeting NLRP3 was investigated. Ox‐LDL‐treated HUVECs were used to reveal the effect of miR‐302c‐3p on pyroptosis as ox‐LDL is a known atherogenic factor that induces endothelial damage and pyroptosis.^15^ The mimic and NC of miR‐302c‐3p were, respectively, transfected into HUVECs which were then treated with ox‐LDL for 24 h. RT‐PCR confirmed that HUVECs treatment with ox‐LDL activated pyroptosis, as demonstrated by increased NLRP3, IL‐1β and caspase‐1 mRNA levels (Figure 3A). Of note, miR‐302c‐3p mimic transfection led to a significant reduction in NLRP3, IL‐1β and caspase‐1 mRNA levels (Figure 3A). The anti‐pyroptosis role of miR‐302c‐3p was also confirmed at the protein level, as reflected by decreased NLRP3, IL‐1β, caspase‐1 and GSDMD protein expression (Figure 3B,C). Addition of miR‐302c‐3p effectively inhibited the release of ox‐LDL‐induced LDH (Figure 3D), and the reduced PI‐positive cells further confirmed the anti‐pyroptosis effect of miR‐302c‐3p overexpression (Figure 3E,F). These data suggested that miR‐302c‐3p overexpression inhibited ox‐LDL‐induced pyroptosis of HUVECs.

### 3.4 Knockdown of miR‐302c‐3p increased ox‐LDL‐induced pyroptosis in HUVECs

**FIGURE 4:**
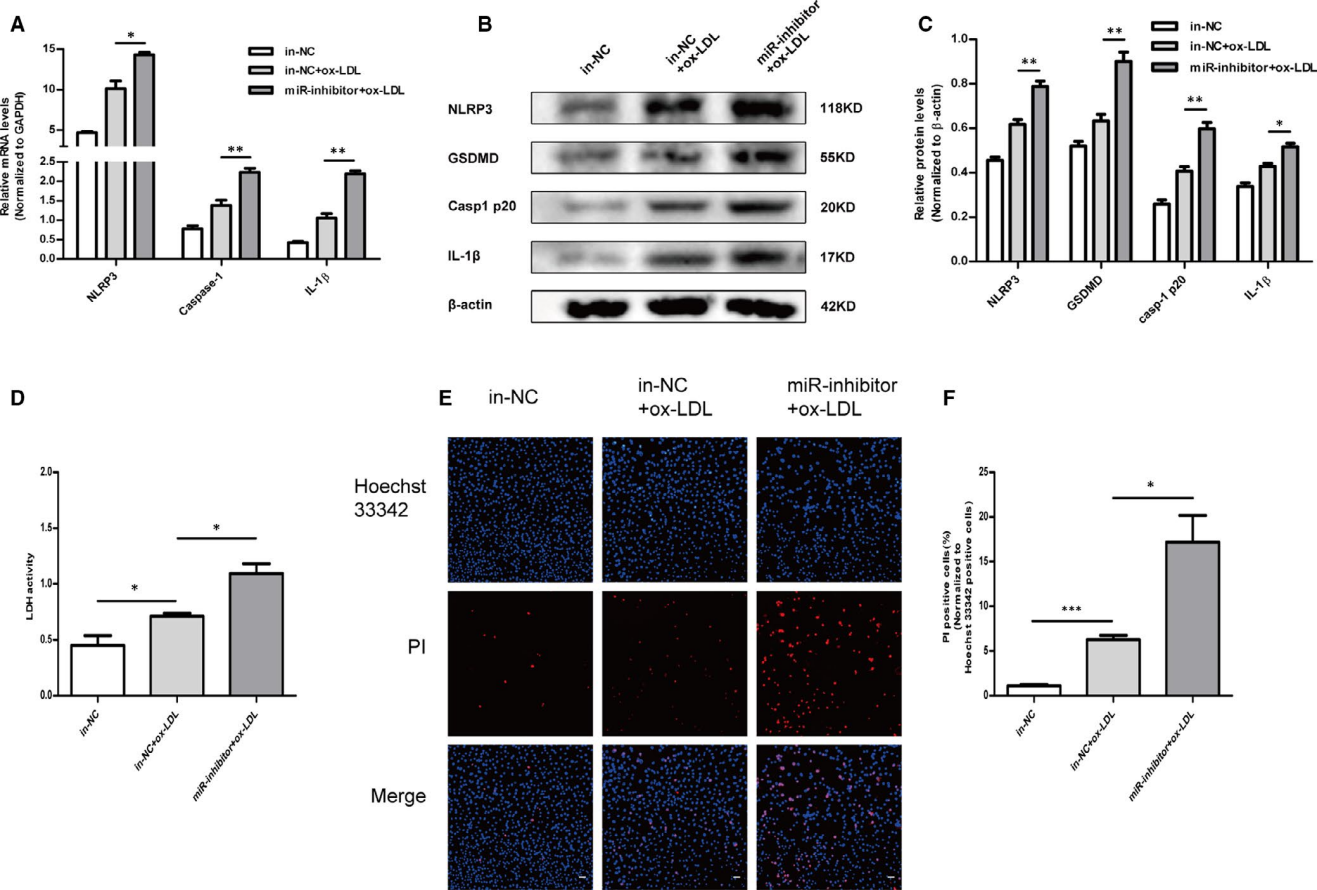
Knockdown of miR‐302c‐3p increases ox‐LDL‐induced pyroptosis in HUVECs. HUVECs were transfected with the miR‐302c‐3p inhibitor negative control (in‐NC) or miR‐302c‐3p inhibitor and then treated with 100 µg/mL ox‐LDL. A, qRT‐PCR analysis of NLRP3, caspase‐1 and IL‐1β expression. B and C, Western blot analysis and quantification of NLRP3, GSDMD, casp1 p20 and IL‐1β expression. D, Lactate dehydrogenase (LDH) release assay detecting LDH activity in the cell supernatant. E, Representative fluorescence micrographs of cells stained with propidium iodide (PI, red) and Hoechst 33 342 (blue). F, Quantification of the percentage of PI‐positive cells. PI‐positive cells are increased in the miR‐302c‐3p inhibitor + ox‐LDL group compared to those in the in‐NC + ox‐LDL group. Scale bars: 50 μm. *P <.05; **P <.01; ***P <.001. Error bars indicate the mean ± SEM of at least triplicate independent experiments

The above results indicated that miR‐302c‐3p overexpression had a protective effect against ox‐LDL‐induced endothelial cell pyroptosis. To further verify whether down‐regulation of endogenous miR‐302c‐3p would aggravate endothelial cell pyroptosis, the inhibitor and inhibitor NC (in‐NC) of miR‐302c‐3p were, respectively, transfected into HUVECs which were then treated with ox‐LDL for 24 h. mRNA levels of NLRP3, IL‐1β and caspase‐1 increased in the miR‐302c‐3p inhibitor transfection group compared to those in the in‐NC group (Figure 4A). Additionally, Western blot results showed increased NLRP3, IL‐1β, caspase‐1 and GSDMD protein expression after transfection with the miR‐302c‐3p inhibitor (Figure 4B,C). Furthermore, the LDH release assay suggested that knockdown of miR‐302c‐3p aggravated endothelial cell pyroptosis in HUVECs treated with ox‐LDL (Figure 4D). These results were further confirmed by increased numbers of PI‐positive cells in the group of transfection with miR‐302c‐3p inhibitor (Figure 4E,F). The above data indicated that down‐regulation of endogenous miR‐302c‐3p increased ox‐LDL‐induced pyroptosis of HUVECs.

### 3.5 Silencing of NLRP3 expression diminished the effect of miR‐302c‐3p on pyroptosis

**FIGURE 5:**
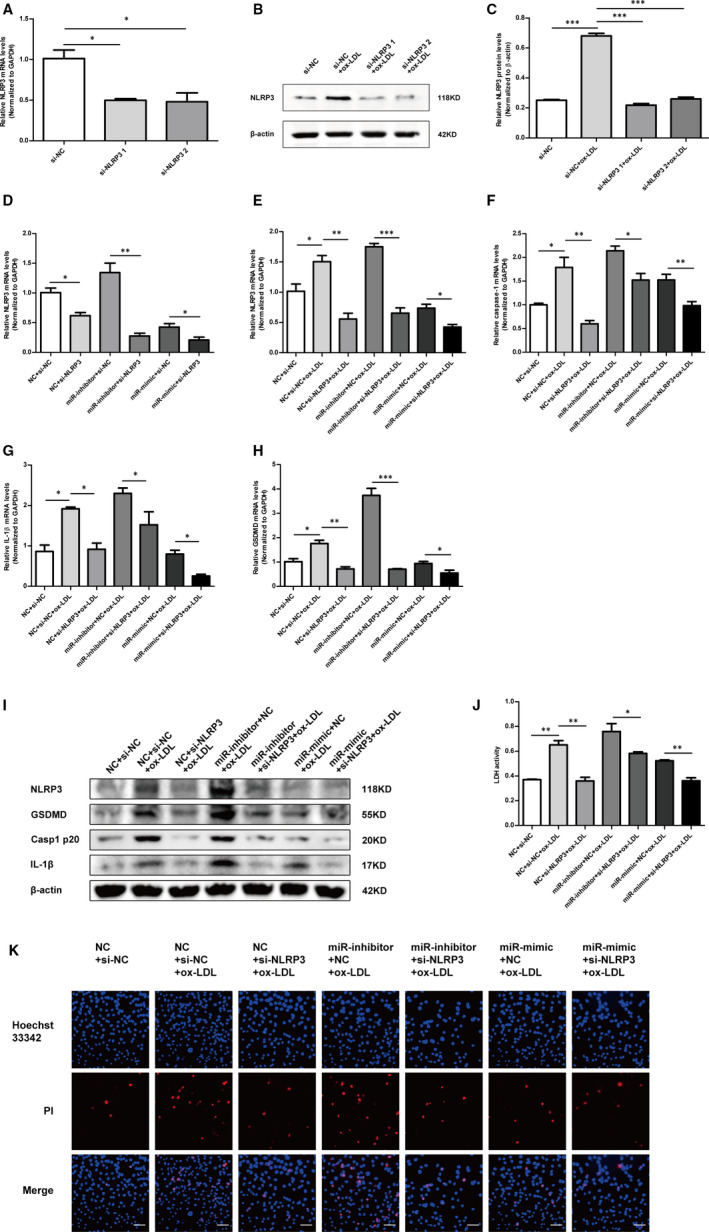
Silencing of NLRP3 expression diminishes the miR‐302c‐3p effect on pyroptosis. A, qRT‐PCR analysis of NLRP3 expression in HUVECs transfected with si‐NC, si‐NLRP3 1 and si‐NLRP3 2, respectively. B and C, Western blot analysis and quantification of NLRP3 expression in HUVECs transfected with si‐NC, si‐NLRP3 1 and si‐NLRP3 2, respectively. D, qRT‐PCR analysis of NLRP3 expression in HUVECs transfected as indicated and untreated with ox‐LDL. E‐H, qRT‐PCR analysis of NLRP3, caspase‐1, GSDMD and IL‐1β expression in HUVECs transfected as indicated and then treated with 100 µg/mL ox‐LDL. I, Western blot analysis of NLRP3, GSDMD, casp1 p20 and IL‐1β expression in HUVECs treated as indicated. J, Lactate dehydrogenase (LDH) release assay detecting LDH activity in the cell supernatant of HUVECs treated as indicated. K, Representative fluorescence micrographs of cells stained with propidium iodide (PI, red) and Hoechst 33 342 (blue). Scale bars: 50 μm. *P < .05; **P < .01; ***P < .001. Error bars indicate the mean ± SEM of at least triplicate independent experiments

To further investigate whether silencing NLRP3 expression might mitigate the effects of miR‐302c‐3p on pyroptosis, two small‐interfering RNAs (siRNAs) were designed to silence NLRP3 expression. RT‐PCR (Figure 5A) and Western blot were performed to verify the transfection efficiency (Figure 5B,C). Si‐NLRP3 1 was selected for follow‐up experiments because of its better inhibitory efficiency. NLRP3 mRNA expression was detected in cotransfection of si‐NLRP3 and miR‐302c‐3p mimic/inhibitor. NLRP3 mRNA expression decreased in the group of cotransfection of miR‐302c‐3p inhibitor and si‐NLRP3 compared to that in the group of cotransfection of miR‐302c‐3p inhibitor and si‐NC. Meanwhile, decreased NLRP3 expression was observed in the group of cotransfection of miR‐302c‐3p mimic and si‐NLRP3 compared to that in the group of cotransfection of miR‐302c‐3p mimic and si‐NC (Figure 5D). These results revealed that silencing NLRP3 expression mitigated the effects of miR‐302c‐3p under physiological conditions without ox‐LDL treatment. In addition, the effects of silencing NLRP3 expression were further demonstrated in HUVECs treated with ox‐LDL. After HUVECs were cotransfected and then treated with ox‐LDL, the mRNA (Figure 5E–H) and protein (Figure 5I) expression of NLRP3, IL‐1β, caspase‐1 and GSDMD were determined. Si‐NLRP3 transfection treatment alleviated the effects of miR‐302c‐3p on pyroptosis. LDH release assay (Figure 5J) and Hoechst 33342/PI staining assay (Figure 5K) demonstrated similar results. Together, the above results established that silencing NLRP3 expression mitigated the effects of miR‐302c‐3p on pyroptosis.

### 3.6 MiR‐302c‐3p up‐regulation inhibited the inflammation and pyroptosis in a mouse model of atherosclerosis

**FIGURE 6:**
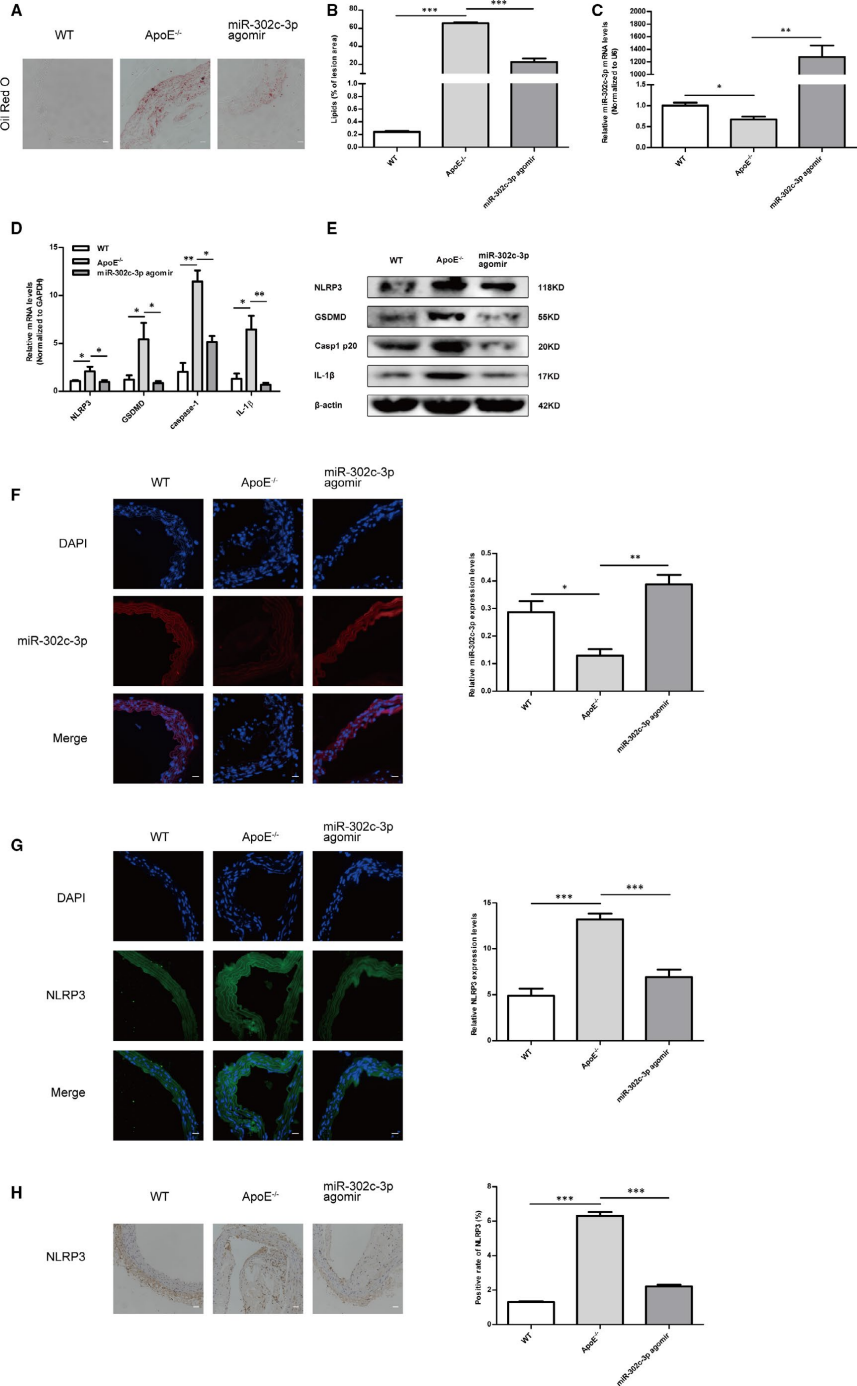
MiR‐302c‐3p up‐regulation inhibits pyroptosis in aortic intima of high‐fat diet (HFD)‐fed ApoE^−/−^ mice. A, Representative images of aortic cross section with Oil red O staining of the wild‐type (WT) group (WT mice with normal diet), ApoE^−/−^ group (ApoE^−/−^ mice with a HFD) and the miR‐302c‐3p agomir group (HFD‐ApoE^−/−^ mice with miR‐302c‐3p agomir treatment). B, Quantification of atherosclerotic lipid area using imageJ. C, qRT‐PCR analysis of miR‐302c‐3p levels in the WT group, ApoE^−/−^ group and miR‐302c‐3p agomir group. D, qRT‐PCR analysis of NLRP3, caspase‐1, GSDMD and IL‐1β expression in the WT group, ApoE^−/−^ group and miR‐302c‐3p agomir group. E, Western blot analysis of NLRP3, GSDMD, casp1 p20 and IL‐1β expression in the WT group, ApoE^−/−^ group and miR‐302c‐3p agomir group. F, miR‐302c‐3p expression detected by FISH in the WT group, ApoE^−/−^ group and miR‐302c‐3p agomir group. G, Immunofluorescence analysis of NLRP3 expression in the WT group, ApoE^−/−^ group and miR‐302c‐3p agomir group. H, Immunohistochemical assay of NLRP3 expression in the WT group, ApoE^−/−^ group and miR‐302c‐3p agomir group. n = 6 in each group. Scale bars: 50 μm. *P < .05; **P < .01; ***P < .001. Error bars indicate the mean ± SEM of at least triplicate independent experiments

Finally, the anti‐pyroptosis effect of miR‐302c‐3p in an in vivo atherosclerosis model was investigated. miR‐302c‐3p agomir was reconstituted with PEI‐*g*‐PEG and injected via the tail vein, according to a previous study.^38^ Oil red O staining of cross section of aorta showed that the atherosclerotic lipids areas significantly increased in the HFD‐fed ApoE^−/−^ group and were reduced in the group of treatment with miR‐302c‐3p agomir (Figure 6A,B). As expected, miR‐302c‐3p was significantly increased in the group of treatment with miR‐302c‐3p agomir in comparison with that in the WT group (Figure 6C). In addition, the mRNA (Figure 6D) and protein (Figure 6E) expression of NLRP3, IL‐1β, caspase‐1 and GSDMD decreased after miR‐302c‐3p agomir treatment compared to those in the HFD‐fed ApoE^−/−^ group. Immunofluorescence analysis also demonstrated that miR‐302c‐3p expression was low in the ApoE^−/−^ group fed with HFD, but highly expressed in the group of treatment with miR‐302c‐3p agomir (Figure 6F). By comparison, NLRP3 expression was inversely related to miR‐302c‐3p expression, which was verified by immunofluorescence (Figure 6G) and immunohistochemical assays (Figure 6H). Overall, these results demonstrated that up‐regulation of miR‐302c‐3p had an anti‐pyroptosis role in vivo.

## 4 DISCUSSION

Chronic inflammation caused by the accumulation of oxidized lipoproteins under the intima is the nature of atherosclerosis.^6^ This concept has promoted the formation of a new promising treatment model, an anti‐inflammatory therapy achieved by inhibiting proinflammatory cytokines, which synergistically delays the process of atherosclerosis with lipid‐lowering therapy.^4^, ^9^, ^39^, ^40^, ^41^ In this regard, inflammasomes have attracted widespread attention, because of their outstanding contribution to the field of immune monitoring and inflammation.^42^ Among them, research on the NLRP3 inflammasome is the most extensive and in‐depth. Therefore, we propose that a powerful regulatory factor may be identified to inhibit the vicious circle of excessive inflammation by inhibiting NLRP3 inflammasome activation, thereby impeding the formation of atherosclerotic plaques. Among the multiple potential regulators upstream of the NLRP3 inflammasome, miRNAs, widely studied non‐coding RNA molecules that silence the expression of downstream mRNA target proteins through translational repression, were investigated.^29^, ^31^, ^32^, ^34^

In this study, miRNAs that directly target NLRP3 were screened. Among these miRNAs, miR‐302c‐3p was a member of the miR‐302/367 cluster and was highly homologous to other members. The miR‐302/367 cluster is closely related to the development of cardiovascular diseases, cancer, neurodegenerative diseases and immunity.^43^ For example, the miR‐302/367 cluster promotes cardiomyocyte proliferation, and overexpression of miR‐302 improves myocardial regeneration and repair after myocardial infarction.^44^ Noticeably, the miR‐302/367 cluster regulates the immune response under external stimuli and prevents excessive inflammation and tissue damage, which is largely related to the nuclear factor (NF)‐κB signalling pathway.^43^ For example, Xiao *et al* have found that miR‐302e limits allergic inflammation by inhibiting the NF‐κB pathway in human mast cell lines.^45^ However, whether the miR‐302/367 cluster was involved in atherosclerosis by regulating the NLRP3 inflammasome and its underlying mechanisms remained unclear. Here, we found that miR‐302c‐3p inhibited the activation of NLRP3 inflammasome and pyroptosis by directly targeting NLRP3 in vivo and in vitro. This unreported function of miR‐302c‐3p provides miR‐302c‐3p with a new regulatory mechanism in the field of cardiovascular inflammation.

A large amount of epidemiological and experimental studies suggests that NLRP3 inflammasome plays a pivotal role in the pathogenesis of atherosclerosis,^11^, ^46^, ^47^, ^48^ driving the discovery of a series of exogenous and endogenous inhibitors that exert anti‐inflammatory effects by inhibiting NLRP3 inflammasome activation or IL‐1 cytokine secretion.^9^, ^49^ For example, clinically approved IL‐1 pharmacological blockers include anakinra, rilonacept and canakinumab, as well as several inhibitors that are in clinical trials, which are introduced in detail in.^9^ Several small molecular inhibitors, such as MCC950, BHB and OLT1177, inhibit NLRP3 inflammasome activation, but are far from clinical application.^50^ Of note, colchicine, a NLRP3 inflammasome inhibitor, has been identified that exerts anti‐inflammatory properties in patients with acute coronary syndrome partly by blocking the activity of monocyte caspase‐1 and thereby inhibiting NLRP3 inflammasome activation.^51^ NcRNAs are a class of novel and promising therapeutic targets in cardiovascular disease.^19^, ^52^, ^53^, ^54^, ^55^, ^56^ In particular, extensive research has been conducted on miRNAs, some of which have entered clinical trials.^57^ Recently, an increasing number of researchers have explored the role of miRNAs in regulating NLRP3 inflammasome, such as miR‐223,^58^, ^59^ miR‐22,^60^, ^61^ miR‐21,^62^, ^63^ miR‐155,^64^, ^65^ miR‐495,^66^ miR‐190^67^ and miR‐30e.^68^ A wide range of diseases is involved, including myocardial ischaemia/reperfusion injury, diabetic cardiomyopathy, acute and chronic liver injury, gastric cancer, endotoxic shock, acute lung injury, Parkinson's disease, neuroinflammation, inflammatory bowel diseases, brain injury and retinopathy. Therefore, miRNAs are considered to have powerful functions in regulating NLRP3 inflammasome‐related diseases and have great potential as therapeutic targets. To achieve its maximum beneficial effects, miRNA therapy needs to ensure that the following conditions are met: stable delivery, high targeting and specificity, and reduction of off‐target effects and side effects. With further research, these problems are being resolved. Despite these challenges, miRNA therapy has broad prospects because of its unique and complex mechanisms regulating the microenvironment that cannot be achieved by drugs.

In summary, these findings are the first to demonstrate that miR‐302c‐3p exerts an anti‐pyroptosis effect in vivo and in vitro by directly targeting NLRP3 and inhibiting NLRP3 inflammasome activation. Intriguingly, this study suggested that miR‐302c‐3p might be a powerful and attractive target for inhibiting endothelial inflammation and pyroptosis in atherosclerosis, providing a novel approach for preventing or alleviating the progression of atherosclerosis.

## CONFLICT OF INTEREST

The authors declare that they have no conflict of interest.

## AUTHOR CONTRIBUTIONS

**Baochen Bai:** Data curation (supporting); Formal analysis (supporting); Investigation (supporting); Methodology (supporting); Resources (supporting). **Yanyan Yang:** Funding acquisition (equal); Supervision (equal); Validation (equal); Visualization (equal); Writing‐review & editing (equal). **Shengxiang Ji:** Conceptualization (supporting); Resources (supporting); Writing‐review & editing (supporting). **Shi‐zhong Wang:** Resources (supporting). **Xingang Peng:** Resources (supporting). **Chao Tian:** Data curation (lead); Formal analysis (lead); Investigation (lead); Methodology (lead); Resources (lead); Software (lead); Writing‐original draft (lead); Writing‐review & editing (lead). **Rui‐Cong Sun:** Investigation (supporting); Methodology (supporting); Resources (supporting). **Tao Yu:** Conceptualization (lead); Funding acquisition (lead); Project administration (equal); Supervision (equal); Validation (equal); Visualization (equal); Writing‐review & editing (equal). **xianming chu:** Conceptualization (equal); Funding acquisition (equal); Project administration (equal); Supervision (equal).

## Supporting information

Figure S1

Table S1‐S2

## Data Availability

The data of this study are available from the corresponding author upon reasonable request.
