# Supplementary material for: MicroRNA‐302c‐3p inhibits endothelial cell pyroptosis via directly targeting NOD‐, LRR‐ and pyrin domain‐containing protein 3 in atherosclerosis
Source: J Cell Mol Med. 2021 Mar 30;25(9):4373–86. doi: 10.1111/jcmm.16500 (PMC8093969; doi:10.1111/jcmm.16500)
Supplement: Supplementary file 1 — Figure S1 [file JCMM-25-4373-s001.pdf]

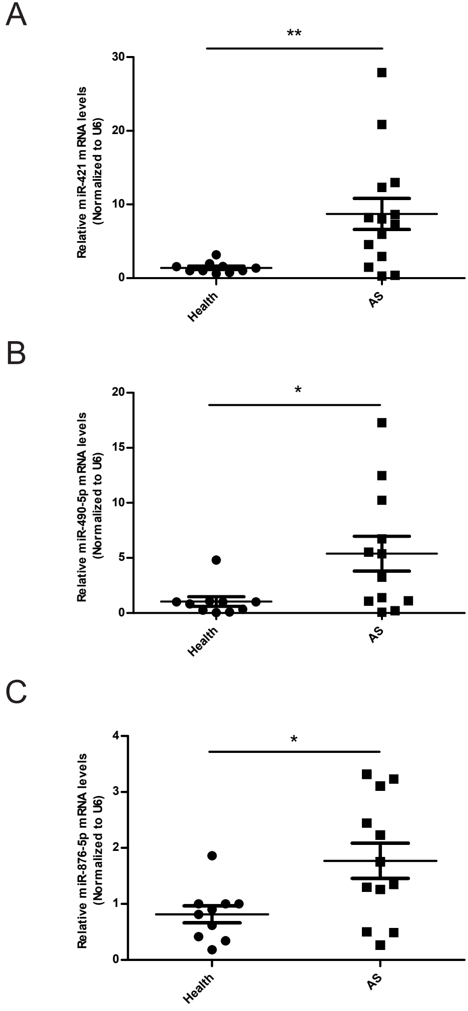
A
**
30
20
10
0
Relative miR-421 mRNA levels (Normalized to U6)
Health
AS
B
*
20
15
10
5
0
Relative miR-490-5p mRNA levels (Normalized to U6)
Health
AS
C
*
4
3
2
1
0
Relative miR-876-5p mRNA levels (Normalized to U6)
Health
AS
