# Supplementary material for: MicroRNA‐302c‐3p inhibits endothelial cell pyroptosis via directly targeting NOD‐, LRR‐ and pyrin domain‐containing protein 3 in atherosclerosis
Source: J Cell Mol Med. 2021 Mar 30;25(9):4373–86. doi: 10.1111/jcmm.16500 (PMC8093969; doi:10.1111/jcmm.16500)
Supplement: Supplementary file 2 — Table S1‐S2 [file JCMM-25-4373-s002.docx]

**Supplementary Table 1. RNA oligo used for transfection in this study.**

| Name | Sequence (5’-3’) |
| --- | --- |
| Hsa-miR-302c-3p mimics | UAAGUGCUUCCAUGUUUCAGUGG |
|  | ACUGAAACAUGGAAGCACUUAUU |
| Hsa-miR-302c-3p NC | UUCUCCGAACGUGUCACGUTT |
|  | ACGUGACACGUUCGGAGAATT |
| Hsa-miR-302c-3p inhibitor | CCACUGAAACAUGGAAGCACUUA |
| Hsa-miR-302c-3p inhibitor NC | CAGUACUUUUGUGUAGUACAA |
| Si-NLRP3-1 | GGAGAGACCUUUAUGAGAATT |
|  | UUCUCAUAAAGGUCUCUCCTT |
| Si-NLRP3-2 | GCAAAGGGCCAUGGACUAUTT |
|  | AUAGUCCAUGGCCCUUUGCTT |
| Si-NC | UUCUCCGAACGUGUCACGUTT |
|  | ACGUGACACGUUCGGAGAATT |
| Hsa-miR-302c-3p-WT | TAAGTGCTTCCATGTTTCAGTGG-biotin |
| Hsa-miR-302c-3p-Mut | TTTCACGAACCATGTTTCAGTGG-biotin |

**Supplementary Table 2. Primers sequence used for qRT-PCR in this study.**

| Gene | Sequence (5’-3’) |
| --- | --- |
| Hsa-NLRP3 | Forward:CAACCTCACGTCACACTGCT |
|  | Reverse:TTTCAGACAACCCCAGGTTC |
| Hsa-caspase-1 | Forward:GCCTGTTCCTGTGATGTGGAG |
|  | Reverse:TGCCCACAGACATTCATACAGTTTC |
| Hsa-IL-1β | Forward:GCGGCATCCAGCTACGAATCT |
|  | Reverse:GGGCAGGGAACCAGCATCTT |
| Hsa-GAPDH | Forward:AAGAAGGTGGTGAAGCAGGC |
|  | Reverse:TCCACCACCCAGTTGCTGTA |
| Mus-NLRP3 | Forward:TGCGATCAACAGGCGAGACCT |
|  | Reverse:CCATCCACTCTTCTTCAAGGCTGTC |
| Mus-caspase-1 | Forward:GATGGCACATTTCCAGGACTGA |
|  | Reverse:TGTTGCAGATAATGAGGGCAAGAC |
| Mus-IL-1β | Forward:GAAATGCCACCTTTTGACAGTG |
|  | Reverse:TGGATGCTCTCATCAGGACAG |
| Mus-GSDMD | Forward:TCTTGGTCGTGGCTCCCGTTGC |
|  | Reverse:ATCGCCTCTGCTGCCGCTTACC |
| Mus-GAPDH | Forward:AAATGGTGAAGGTCGGTGTGAAC |
|  | Reverse:CAACAATCTCCACTTTGCCACTG |
| U6 | Forward:GGAACGATACAGAGAAGATTAGC |
|  | Reverse:TGGAACGCTTCACGAATTTGCG |
| Hsa-miR-302c-3p | TAAGTGCTTCCATGTTTCAGTGG |
| Hsa-miR-490-5p | CCATGGATCTCCAGGTGGGT |
| Hsa-miR-421 | ATCAACAGACATTAATTGGGCGC |
| Hsa-miR-876-5p | TGGATTTCTTTGTGAATCACCA |
